# The Primary Seat of Infection in Tuberculosis

**Published:** 1904-04-30

**Authors:** 


### THE PRIMARY SEAT OF INFECTION IN TUBERCULOSIS.

Much controversy has raged round the question of how the tubercle bacillus commonly gains entrance into the human body. The two chief theories on the subject hold respectively that the usual portal is the respiratory system and the alimentary system, while a third party considers that the lymphoid tissue of the naso-pharynx is commonly the primary nidus. Though it is quite true that any one of these localities may be the first to become infected, the important question is still outstanding as to which is the most frequent primary source of trouble. Drs. J. O. Symes and Theodore Fisher[1] have made an investigation into the matter, analysing the post-mortem reports of 500 fatal cases of tuberculosis in the hospitals of Bristol. As they point out there are several sources of fallacy to be avoided in an examination such as they have carried out. Thus the discovery of disease in a certain part of the body does not necessarily imply that the cause of the disease reached that spot by the nearest route. There is a want of resistance in certain organs and the infecting agent may pass through more resistant parts of the body to invade these weaker organs. Thus they have found acute tuberculosis limited to the peritoneum when the only old focus in the body that could be discovered was a caseous bronchial gland; and they further point out that, as with the mesenteric glands, there is no doubt that the bronchial glands can become infected without the presence of any visible lesion of the lungs or bronchi. Again, if the matter were sufficiently simple to be settled by the evidence afforded by the situation of the oldest foci of disease in the body, there is still the difficulty in many cases of deciding which are the oldest foci. Cases of tuberculosis in children in which the disease is entirely limited to one cavity of the body are considerably in the minority, and the liability to fallacy of personal judgment cannot but influence the decision as to which cavity was first attacked.

In drawing their conclusions the authors have been careful to classify all those cases as doubtful where there would be the least possibility of difference of opinion, although where there are intestinal ulcers, and a few caseous mesenteric glands of apparently recent origin, should there be old pulmonary lesions such as a cavity and perhaps also large masses of caseous bronchial and mediastinal glands, they have generally considered it justifiable to classify the abdominal disease as produced by swallowed sputum infected with tubercle bacilli, and therefore secondary to the disease in the thorax. In 102 cases of tuberculosis in subjects under 12 years of age the site of the original focus was as follows :—definitely abdominal, 11·7 per cent.; definitely thoracic, 55·8 per cent.; doubtful whether abdominal or thoracic 23·5 per cent; other situations, *e.g.* bones, skin, etc., 8·8 per cent. That is to say that the proportion of abdominal to thoracic cases was 1 : 4·7. Subjects under two years of age showed, however, a much higher proportion of abdominal cases. Curiously enough the proportion of definitely abdominal to definitely thoracic cases appeared to be almost as great in the second twelve years of life as in the first twelve years, though after the age of 24 the proportion of abdominal cases greatly decreased. It is probable, as the authors point out, that their percentages cannot be taken to represent the exact relative frequency of abdominal infection, as cases of abdominal tuberculosis are more likely to gain admittance to hospitals than are those where the lungs are principally affected, and the ratio of the former to the latter is therefore probably not so high as their statistics would at first sight seem to indicate.

[1] Brit. Med. Jour., April 16.

